# Incidence of ischemic complications and technical nuances of arteries preservation for insular gliomas resection

**DOI:** 10.3389/fsurg.2022.956872

**Published:** 2022-10-14

**Authors:** Zonggang Hou, Zhenxing Huang, Zhenye Li, Zhenghai Deng, Gen Li, Yaokai Xu, Mingran Wang, Shengjun Sun, Yazhuo Zhang, Hui Qiao, Jian Xie

**Affiliations:** ^1^ Department of Neurosurgery, Beijing Tiantan Hospital, Capital Medical University, Beijing, China; ^2^ Department of Neurophysiology, Beijing Neurosurgical Institute, Capital Medical University, Beijing, China; ^3^ Neuroimaging Center, Beijing Tiantan Hospital, Capital Medical University, Beijing, China; ^4^ Beijing Neurosurgical Institute, Capital Medical University, Beijing, China

**Keywords:** insular gliomas, surgery, middle cerebral artery, lateral lenticulostriate arteries, ischemia, motor evoked potentials, surgical technique

## Abstract

**Introduction:**

Insular gliomas have complex anatomy and microvascular supply that make resection difficult. Furthermore, resection of insular glioma is associated with a significant risk of postoperative ischemic complications. Thus, this study aimed to assess the incidence of ischemic complications related to insular glioma resection, determine its risk factors, and describe a single surgeon’s experience of artery-preserving tumor resection.

**Methods:**

We enrolled 75 consecutive patients with insular gliomas who underwent transcortical tumor resection. Preoperative and postoperative demographic, clinical, radiological [including diffusion-weighted imaging (DWI)], intraoperative neurophysiological data, and functional outcomes were analyzed. Motor evoked potentials (MEPs) and radiological characteristics like the relationship between the proximal segment of the lateral lenticulostriate arteries (LLSAs) and the tumor, the flat inner edge sign (the inner edge of the insular glioma is well-defined) or obscure inner edge sign, the distance between the lesion and posterior limb of the internal capsule and the invasion of the superior limiting sulcus by the tumor were analyzed. Strategies such as “residual triangle,” “basal ganglia outline reappearance,” and “sculpting” technique were used to preserve the LLSAs and the main branches of M2 for maximal tumor resection according to the Berger–Sinai classification.

**Results:**

Postoperative DWI showed acute ischemia in 44 patients (58.7%). Moreover, nine patients (12%) had developed new motor deficits, as determined by the treating neurosurgeons. The flat inner edge sign [odds ratio (OR), 0.144; 95% confidence interval (CI), 0.024–0.876) and MEPs (>50%) (OR, 18.182; 95% CI, 3.311–100.00) were significantly associated with postoperative core ischemia, which affected the posterior limb of the internal capsule or corona radiata.

**Conclusions:**

Insular glioma resection was associated with a high incidence of ischemia, as detected by DWI, as well as new motor deficits that were determined by the treating neurosurgeons. Insular glioma patients with obscure inner edge signs and intraoperative MEPs decline >50% had a higher risk of developing core ischemia. With our strategies, maximal safe resection of insular gliomas may be achieved.

## Introduction

The insular lobe is a common site of intrinsic brain tumor growth (1). Improvements in understanding the insular anatomy and the application of various technologies have made insular glioma operable with acceptable morbidity. Postoperative motor deficits remain a significant concern (2), and ischemia's incidence and risk factors after surgery for insular gliomas need further exploration.

For insular tumors, ischemic strokes can arise by lateral lenticulostriate arteries (LLSAs) from M_1_ segment of the middle cerebral artery (MCA), long insular artery from the M_2_–M_3_ junction at the level of the superior insular sulcus, and even long medullary arteries terminating the M_4_ branches (3–5). Although transitory clipping of the latter two types of arteries under motor evoked potentials (MEPs) monitoring may be helpful (3, 6), the characteristics of the thin, long course and ambiguous origin of these two arteries make their reservation still difficult intraoperatively.

LLSAs supply the internal capsule and often pass along the inner side of insular tumors (7). Although the number of LLSAs varies from 1 to 21, even the occlusion of one branch may cause extensive infarction of the subcortical ganglia and internal capsule, resulting in motor and language deficits (8). Immediate postoperative deficits may be caused by resection-induced contusion, edema, and hypoperfusion. However, permanent deficits are mainly caused by infarctions due to disruption of MCA branches on the lateral surface of the insula and LLSAs within the anterior perforated substance on its mesial side (9, 10). In the transsylvian and transcortical approaches for insular glioma resection, the preservation of these critical blood vessels is essential (11). The characteristics of the relatively large diameter and exact origin of LLSAs, make its reservation possible intraoperatively.

This study tried to determine the incidence of ischemic complications and their risk factors, and describe the strategies which focus on LLSAs and MCA preservation employed to prevent the ischemic complications of insular glioma surgery.

## Methods

### Patient selection

We conducted a single-center, retrospective, noncontrolled study involving 75 insular glioma patients admitted to our department between October 2018 and June 2020. The preoperative and postoperative clinical, radiological, and intraoperative neuromonitoring (IONM) data were analyzed. The Berger–Sanai insular glioma classification was used for tumor classification (12). All tumors were histologically examined and classified according to the World Health Organization (WHO) primary central nervous system tumor classification. This study was approved by our institutional review board.

### Surgical technique

All surgical procedures were performed *via* the transcortical approach under general anesthesia, as previously described (13). All surgical procedures were performed under continuous IONM. In these patients, the transcortical windows were limited to the anterior temporal lobe and the pars orbitalis, triangularis of the inferior frontal gyrus, which rarely involved Broca's area, especially for tumors in the dominant hemispheres (10).

LLSAs were subdivided into two segments: (i) proximal segment, which is anterior to the point of entry into the anterior perforated substance, and (ii) distal segment, which is located after the entry point into the anterior perforated substance (14).

We observed our three critical points in the preservation of LLSAs and the main M2 branches of the MCA.

**Figure 1:**
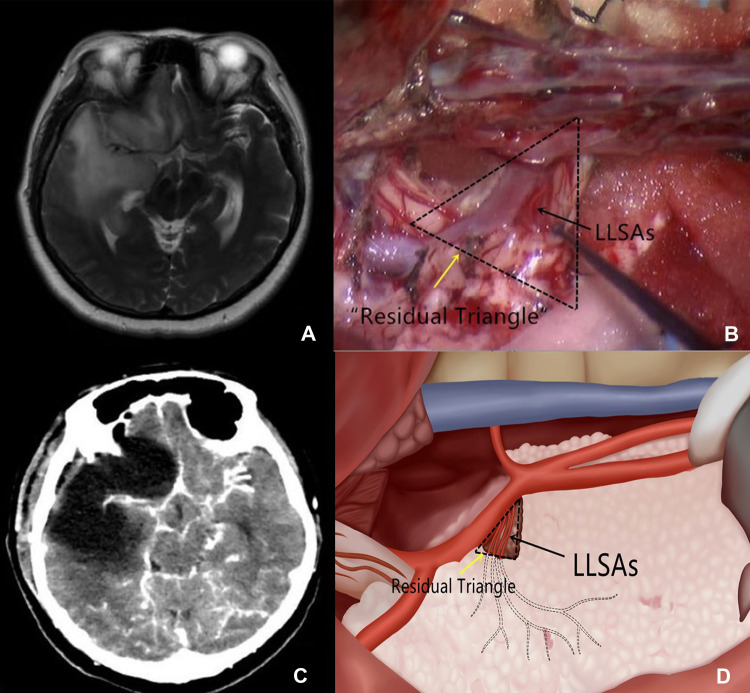
(**A**) T2-weighted imaging shows that the proximal segment of the LLSAs is encased by the glioma. (**B**) Intraoperative imaging shows that a small cone-like tumor tissue at the proximal segment of the LLSAs (black arrow) is retained. It also outlines the residual triangle (dotted triangle, yellow arrow). (**C**) Postoperative CT demonstrates the cone-like tumor tissue supporting the MCA. (**D**) Schematic depiction of the residual triangles and LLSAs. LLSAs, lateral lenticulostriate arteries; CT, computed tomography; MCA, middle cerebral artery.

Intraoperatively, we first attempted to identify the origin of LLSAs at the horizontal branch of M_1_ of the MCA. This procedure was conducted primarily for tumors located in zones I and IV. If the proximal segment of the LLSAs was encased by the tumor (Figure 1A), a small cone-like tumor tissue (Figure 1B) was retained at the level of the anterior perforated substance to preserve the proximal branches of the LLSAs as well as to support the LLSAs and avoid distortion after tumor resection, which could subsequently result in ischemia. The cone-like tumor tissue resembled the shape of a triangle in postoperative imaging; thus, it was termed the “residual triangle” (Figure 1C). The horizontal branch of M_1_ formed the anterior side of the “residual triangle.” The posterior side extended from the origin of the LLSAs to the entry point of the anterior perforated substance. The superior point was at the origin of the LLSAs from M_1_ (Figure 1D).

**Figure 2:**
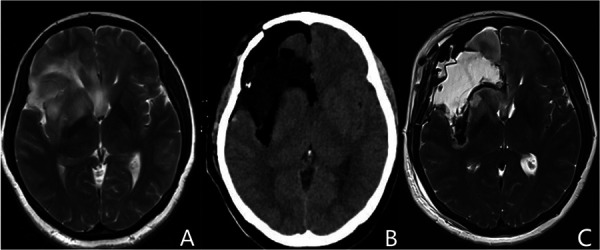
Representative images of the basal ganglia outline reappearance. (**A**) Preoperative MRI shows that the basal ganglia is invaded by insular gliomas. (**B,C**) Postoperative CT and MRI T2 imaging demonstrate the artificial profile of the basal ganglia, which is called the basal ganglia outline reappearance. MRI, magnetic resonance imaging; CT, computed tomography.

Identifying the depth of resection and preserving the distal segment of the LLSAs was another critical point we observed for insular glioma resection. The basal ganglia was usually adjacent to or even invaded by insular gliomas in some cases (Figure 2A). Particularly for tumors that invaded the basal ganglia, the anatomical depth of resection of the basal ganglia (lateral surface of the putamen and the head of the caudate nucleus) (Figure 2B) was dependent on the texture of the basal ganglia and distal branches of the LLSAs. The basal ganglia was orange-yellow and partially crisp in appearance, which was similar to the Chinese tofu kasu and Indonesian nutmeg (15). Intraoperatively, once the structure like this was observed under the microscope, the depth of resection arrived, then the operation continued shallowly to protect the external capsule on the surface of basal ganglia and the distal branches of the LLSA. Then the tumor resection continued at this depth with repeated confirmation of putamen and LLSAs. Thus, the lateral outline of basal ganglia was delineated and termed the “basal ganglia outline reappearance.” Using this method, we prevented direct injury to the pyramidal tract, preserved the distal branches of the LLSA, and safely achieved maximal resection of the tumor.

Finally, as for the insular glioma, insular arteries are the primary providers of blood supply to insular gliomas. The tumor is resected in a subpial fashion with preservation of M_2_ vessels and coagulation of the insular artery. We used initiative rather than passive hemostasis (15). Like a sculptor, the surgeon needs carefulness and patience. This skill was named as “sculpting” technique. After resecting the tumor, the skeletonized main branches of M_2_ were preserved and suspended in the operative cavity (13).

### Radiological data

**Figure 3:**
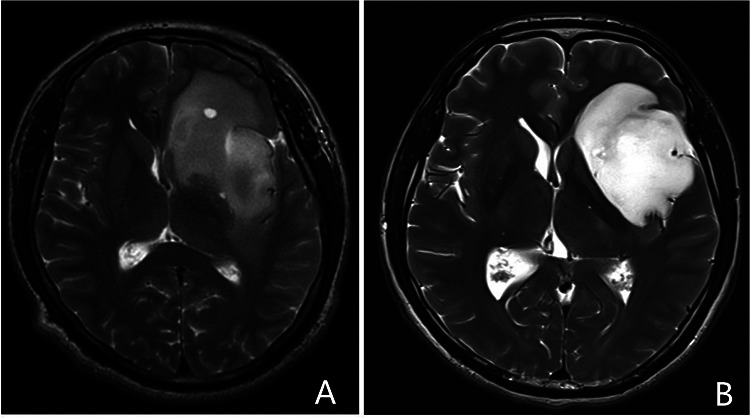
Representative images of the flat inner edge sign of insular gliomas based on the preoperative MRI. (**A**) Insular glioma with an obscure inner edge sign. (**B**) Insular glioma with a flat inner sign. MRI, magnetic resonance imaging.

All patients underwent preoperative and postoperative magnetic resonance imaging (MRI). Postoperative MRI, including diffusion-weighted imaging (DWI), was performed within 72 h after surgery. Evaluation of the imaging was conducted independently by a neuroradiologist and neurosurgeon, who were blinded to the patient's clinical courses. Four radiological characteristics which might associate with ischemia were evaluated. The relationship between the proximal segment of the LLSAs and the tumor was determined on T2-weighted images. Based on this, we decided whether the proximal segment of the LLSAs was encased by the tumor (Figure 1A). The flat inner edge sign used to describe the inner edge of the insular glioma is well-defined without putamen being involved and can be seen on preoperative T2-weighted images. Figure 3, on the contrary, is the obscure inner edge sign. At the level of the foramen of Monro on axial T2-weighted images, a perpendicular line at the midpoint of the posterior limb of the internal capsule was created, and the length of the line to the tumor was defined as the distance between the lesion and posterior limb of the internal capsule. The tumor's invasion of the superior limiting sulcus was identified *via* sagittal and coronal MRI. The volume and extent of resection (EOR) were calculated as previously described (13).

### Intraoperative neuromonitoring

Two experienced technicians performed IONM. Somatosensory evoked potentials (SSEPs) and continued transcranial MEPs of the extremities were monitored in all patients as previously described (16, 17). We defined a decline in MEP amplitude >50% as significant deterioration, provided that technical issues did not cause the decline.

### Postoperative ischemia on MRI and associated neurological outcomes

**Figure 4:**
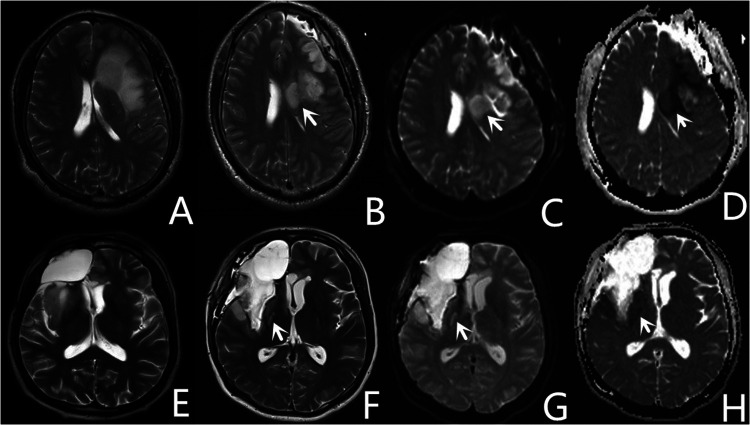
Representative images of two patients with core ischemia. One patient with postoperative ischemia (white arrow) located in the corona radiata (**A–D**), appears as hyperintense images on diffusion-weighted imaging (**C**), and hypointense images on apparent diffusion coefficient (**D**). Another patient with postoperative ischemia (white arrow) located in the posterior limb of the internal capsule (**E–H**).

Areas that appeared hyperintense on DWI and hypointense on apparent diffusion coefficient mapping of early postoperative MRI (Figure 4) were classified as ischemic lesions (4, 18). Diffusion restrictions caused by methemoglobin as identified on T2-weighted gradient echo images were excluded.

The ischemic lesions were further classified into two categories: core ischemia and noncore ischemia. Core ischemia was characterized by circumscribed areas located in the corona radiata (Figures 4B–D) or the posterior limb of the internal capsule (Figures 4F–H). Ischemia in locations other than the corona radiata and the posterior limb of the internal capsule was classified as noncore ischemia.

Postoperative neurological functions, which mainly include motor and language functions, were assessed by two neurosurgeons (ZoH and ZD, with 10 and 15 years of experience, respectively). Next, the data were extracted by ZhH from the medical records and follow-up databases. Any newly developed neurological deterioration was considered a postoperative neurological deficit and evaluated 7 days and 6 months after surgery. Motor function was assessed according to the standardized Medical Research Council (MRC) score.

### Statistical analyses

All statistical analyses were performed using the SPSS for Windows software (version 21.0; IBM Corporation). Significance was set at a *P* value < 0.05 for the entire analysis. The Kolmogorov–Smirnov and equal variance tests were performed before any other statistical analysis. Continuous data with normal distribution were analyzed using Student's *t*-test and expressed as mean ± standard deviation. Data with non-normal distribution were analyzed using the Wilcoxon two-sample test and expressed as median (interquartile range). Categorical data were analyzed using Fischer's exact or chi-square tests. Multivariate logistic regression analysis was used to determine the risk factors for the development of core ischemia. To assess the relationship between the four surgical indicators and outcomes of the surgical procedure, we performed logistic multivariate regression analyses. Any variable with a *P* value < 0.10 in the univariate analysis was considered a potential confounder and further included in the multivariate logistic regression analysis for adjustment.

## Results

### Clinical data, demographic data, and outcomes

A total of 75 patients were included in this study (Table 1). The mean age of the patients was 42.6 ± 12.1 years, and 51 patients (68.0%) were male. Two patients underwent surgery due to tumor recurrence. Moreover, 41 patients (54.7%) had right-sided tumors. Preoperative clinical manifestations included seizures (58.7%), incidental findings (20.0%), limb numbness (14.7%), headache (4.0%), and speech deficits (2.6%). A majority of patients had preoperative Karnofsky Performance Scale (KPS) scores ≥ 90 (93.3%) and modified Rankin Scale score < 2 (94.7%). Based on the Berger–Sanai classification (Table 2), 18 (24.0%) tumors were located in zone I, 3 (4.0%) in zone II, 1 (1.3%) in zone III, 1 (1.3%) in zone IV, 8 (10.7%) in zone I + II, 13 (17.3%) in zone I + IV, 3 (4.0%) in zone II + III, and 11 (14.7%) in zone III + IV. Seventeen tumors (22.7%) were classified as giant.

**Table 1:** Comparison of baseline demographics and clinical characteristics of patients with and without core ischemia and paralysis.

Variable	Total	Ischemia		*P* value	Motor function		*P* value
		No-core ischemia and no ischemia	Core ischemia		No paralysis	Paralysis	
	75	64	11		66	9	
Age	42.6 ± 12.1	41.9 ± 12.5	46.5 ± 8.7	0.147	41.8 ± 12.4	48.4 ± 7.8	0.11
≥55	13	12 (18.8)	1 (9.1)	0.434	12 (18.2)	1 (11.1)	0.599
<55	62	52 (81.3)	10 (90.9)		54 (81.8)	8 (88.9)	
Sex							
Male	51 (68.0)	44 (68.8)	7 (63.2)	0.737	46 (69.7)	5 (55.6)	0.46
Female	24 (32.0)	20 (31.3)	4 (36.4)		20 (30.3)	4 (44.4)	
PA							
2	47 (62.7)	41 (64.1)	6 (54.5)	0.547	42 (63.6)	5 (54.5)	0.72
3 or 4	28 (37.3)	23 (35.9)	5 (45.5)		24 (36.4)	4 (44.4)	
2 or 3	65	55 (85.9)	10 (90.9)	0.654	57 (86.4)	8 (88.9)	0.834
4	10	9 (14.1)	1 (9.1)		9 (13.6)	1 (11.1)	
R-L							
L	34 (45.3)	29 (45.3)	5 (45.5)	0.993	30 (45.5)	4 (44.4)	1
R	41 (54.7)	35 (54.7)	6 (54.5)		36 (54.5)	5 (55.6)	
Flat inner edge sign							
Yes	47 (62.7)	45 (70.3)	2 (18.2)	0.001^a^	46 (69.7)	1 (11.1)	0.001^a^
No	28 (37.3)	19 (29.7)	9 (81.8)		20 (30.3)	8 (88.9)	
Enhancement							
Yes	22 (29.3)	16 (25.0)	6 (54.5)	0.047^a^	17 (25.8)	5 (55.6)	0.113
No	53 (70.7)	48 (75.0)	5 (45.5)		49 (74.2)	4 (44.4)	
Intact superior limiting sulcus							
Yes	45 (60.0)	41 (64.1)	4 (36.4)	0.083	43 (65.2)	2 (22.2)	0.025^a^
No	30 (40.0)	23 (35.9)	7 (63.6)		23 (34.8)	7 (77.8)	
Encased of initial of LLSAs							
Yes	51 (68.0)	45 (70.3)	6 (54.5)	0.3	46 (69.7)	5 (55.6)	0.455
No	24 (32.0)	19 (29.7)	5 (45.5)		20 (30.3)	4 (44.4)	
Distance to the posterior limb (mm)	4.40 ± 5.05	4.93 ± 4.68	1.96 ± 6.51	0.972	5.11 ± 5.09	0 ± 0	0.002^a^
Preop tumor vol (cm^3^)	45.32 (25.99, 77.89)	45.16 (24.56, 79.90)	54.87 (25.99, 64.66)	0.869	45.16 (24.02, 78.81)	56.62 (28.37, 70.36)	0.744
Hypertension							
Yes	13 (17.3)	10 (15.6)	3 (27.3)	0.346	10 (15.2)	3 (33.3)	0.183
No	62 (82.7)	54 (84.4)	8 (72.7)		56 (84.8)	6 (66.7)	
Diabetes mellitus							
Yes	6 (8.0)	4 (6.3)	2 (18.2)	0.178	4 (6.1)	2 (22.2)	0.149
No	69 (92.0)	60 (93.8)	9 (81.8)		62 (93.9)	7 (77.8)	
Smoking							
Yes	27 (36.0)	23 (35.9)	4 (36.4)	0.978	23 (34.8)	4 (44.4)	0.714
No	48 (64.0)	41 (64.1)	7 (63.6)		43 (65.2)	5 (55.6)	
Preop KPS							
≥90	70 (93.3)	61 (95.3)	9 (81.8)	0.097	63 (95.5)	7 (77.8)	0.106
<90	5 (6.7)	3 (4.7)	2 (18.2)		3 (4.5)	2 (22.2)	
Preop mRS							
<2	72 (96.0)	61 (95.3)	11 (10.0)	0.464	63 (95.5)	9 (100)	0.2
≥2	3 (4.0)	3 (4.7)	0 (0.0)		3 (4.5)	0 (0.0)	
BMI in kg/m^2^	23.9 ± 3.0	24.1 ± 3.1	22.7 ± 2.4	0.24	24.2 ± 3.1	22.1 ± 2.0	0.137
MEP							
Normal	64 (85.3)	60 (93.8)	4 (36.4)	<0.001^a^	62 (93.9)	2 (22.2)	<0.001^a^
Abnormal	11 (14.7)	4 (6.3)	7 (63.6)		4 (6.1)	7 (77.8)	
EOR							
≧90%	56 (74.7)	49 (76.6)	7 (63.6)	0.363	50 (75.8)	6 (66.7)	0.684
<90%	19 (25.3)	15 (23.4)	4 (36.4)		16 (24.2)	3 (33.3)	
IDH							
Yes	60 (81.1)	51 (81.0)	9 (81.8)	0.946	53 (81.5)	7 (77.8)	0.676
No	14 (18.9)	12 (19.0)	2 (18.2)		12 (18.5)	2 (22.2)	
1p/19q							
Yes	22 (31.9)	19 (31.1)	3 (37.5)	0.717	19 (30.6)	3 (42.9)	0.672
No	47 (68.1)	42 (68.9)	5 (62.5)		43 (69.4)	4 (57.1)	
MGMT							
Yes	49 (71.0)	42 (68.9)	7 (87.5)	0.274	42 (67.7)	7 (100.0)	0.98
No	20 (29.0)	19 (31.1)	1 (12.5)		20 (32.3)	0 (0.0)	

PA, pathology; LLSAs, lateral lenticulostriate arteries; KPS, Karnofsky performance status; mRS, modified Rankin scale; BMI, body mass index; MEP, motor evoked potentials; EOR, extent of resection; IDH, isocitrate dehydrogenase; MGMT, O6-methylguanine-DNA methyltransferase.

^a^ These values are significant at statistical analysis.

**Table 2:** Summary of resected insular gliomas by zone.

Zone	WHO grade			IDH	Volume (cm^3^)	No ischemia	No-core ischemia	Core ischemia	Residual triangle	Paralysis
	II	III	IV							
I (*n* = 18)	14	2	2	16	32.29	7	10	1	8	0
II (*n* = 3)	2	1	0	3	83.1	1	0	2	0	2
III (*n* = 1)	0	0	1	1	39.38	0	1	0	1	0
IV (*n* = 1)	0	0	1	0	66.13	1	0	0	0	0
I + II (*n* = 8)	5	1	2	6	48.15	2	6	0	5	0
I + IV (*n* = 13)	10	2	1	12	51.16	7	4	2	8	1
II + III (*n* = 3)	1	1	1	1	61.22	0	2	1	1	1
III + IV (*n* = 11)	4	5	2	6	59.74	5	4	2	7	2
Giant (*n* = 17)	10	5	2	15	88.41	8	6	3	13	3
Totals	46	17	12	60		31	33	11	43	9

IDH, isocitrate dehydrogenase; WHO, World Health Organization.

The mean preoperative tumor volume was 57.7 ± 43.4 cm^3^. The EOR was ≥90% in 56 patients (74.6%). There was no significant difference in the preoperative tumor volume (*P* > 0.05) or EOR (*P* > 0.05) between low-grade gliomas (LGGs) and high-grade gliomas (HGGs). The mean EOR was 91.4% in zone I, 86.3% in zone II, 94.0% in zone III, 100% in zone IV, 90.5% in zone I + II, 89.4% in zone I + IV, 92% in zone II + III, 89.1% in zone III + IV, and 89.7% in giant tumors.

The histological composition of tumors was as follows: WHO grade II (62.7%) glioma in 47 patients, grade III glioma (24.0%) in 18 patients, and grade IV glioma (13.3%) in 10 patients. Molecular diagnostic tests revealed the presence of isocitrate dehydrogenase 1/2 mutations in 60 of the 74 cases (71.1%), 1p19q co-deletion in 22 of the 69 cases (31.9%), and O^6^-methylguanine-DNA methyltransferase promoter methylation in 49 of the 69 cases (71.1%).

Radiotherapy or chemotherapy was recommended postoperatively based on molecular biomarkers and EOR for LGGs. For HGGs, concurrent chemoradiotherapy was performed following the Stupp regimen (19).

Complete short-term and long-term (within 7 days and 6 months postoperatively, respectively) follow-up data were available for all 75 patients. Postoperatively, at 7 days, motor deficits were observed in nine patients (12.0%, according to the Standardized MRC score 3–4/5 in six cases and 0–2/5 in three cases). Facial weakness occurred in six patients (8%). At the 6 months follow-up, two of the nine patients with an early postoperative mild motor deficit demonstrated full recovery, and the other seven patients also improved—five of the seven patients were able to walk (MRC score 4/5, lower limb; MRC score 3–4/5, upper limb), and the remaining two patients unable to walk (MRC score 3/5, both lower and upper limbs). Additionally, facial weakness resolved in five patients with facial. Moreover, the sixth patient with facial weakness also showed improvement.

Language deficits were found in 8 of the 34 patients who underwent surgery on their dominant side, and 4 of 8 patients with aphasia recovered within 7 days. At the 6 month follow-up, improvement in language function was observed in the other four patients with aphasia. However, mild speech fluency disorders persisted in three of these four patients, and mild impairment in speech comprehension stayed in one of these patients.

### Risk factors of core ischemia and paralysis

Of the 75 patients, 44 (58.7%) presented with acute ischemia as detected by postoperative DWI, most of which were located below the resection cavity. Further, the size varied on a case-to-case basis. The median infarction volume was 7.77 cm^3^ (range 0.69–27.76). The following locations were involved by stroke: ischemia surrounding the resection cavity, 26 (59.1%); the deep-seated ischemia, including the caudate nucleus, corona radiata, and internal capsule, 5 (11.4%); the combination of the latter two, 13 (29.5%). Of the 18 ischemia involving deep structure, head of caudate nucleus and anterior limb of internal capsule ischemia in seven (38.9%) patients, caused by the anterior branch of LLSAs; posterior limb of internal capsule ischemia in three (16.7%) patients, caused by the posterior branch LLSAs; and corona radiata ischemia in eight (44.4%) patients, caused by the long insular artery. The latter 11 patients were determined as core ischemia. Nine of the 11 patients with core ischemia developed new acute motor deficits (Table 1). We also found that six of 11 patients with core ischemia and six of nine patients with paralysis had tumor locations related to zone II.

The retrospective analysis of preoperative MR images revealed the presence of flat inner edge signs in 47 of the 75 tumors (62.7%). Core ischemia developed in two patients, and motor complications developed in one of the 47 patients with flat inner edge signs, which was significantly lower than the number of patients with obscure inner edge signs who developed motor complications (*P* < 0.05).

The tumor encased the proximal segment of the LLSAs in 51 of the 75 cases. The “residual triangle” was found in 43 patients, accounting for 84.3% of the 51 patients with encased tumors. The “residual triangle” volume ranged from 0.38 to 8.89 cm^3^, with a mean value of 1.06 cm^3^, accounting for 1.7% of the preoperative tumor volume. Evidence of the superior limiting sulcus invaded by the tumor was identified in 30 of the 75 patients (40.0%). The distance between the lesion and the posterior limb of the internal capsule was 4.50 ± 5.05 mm. None of these three neuroimaging factors were significantly associated with core ischemia.

IONM data of all 75 patients were available. Eleven patients (14.7%) showed a more than 50% decline in MEP intraoperatively. The decline in MEP was significantly associated with the presence of core ischemia (*P *< 0.05), postoperative paralysis (*P* < 0.05), and paralysis at the 6 month follow-up (*P* < 0.05), but not with noncore ischemia (*P* > 0.05).

After a multivariate analysis of potential risk factors for core ischemia, flat inner edge sign [odds ratio (OR), 0.144; 95% confidence interval (CI), 0.024–0.876) and MEPs (<50%) (OR, 18.182; 95% CI, 3.311–100.00) had significant associations with postoperative core ischemia (Table 3). Additionally, another multivariate analysis of potential risk factors for paralysis revealed that flat inner edge sign (OR, 0.079; 95% CI, 0.007–0.914) and MEPs (<50%) (OR, 40.00; 95% CI, 5.24–333.33) had significant associations with postoperative paralysis (Table 4).

**Table 3:** Multivariate logistic regression analyses for estimating risk factors for core ischemia.

Variables	Unadjusted OR	*P* value	Adjusted OR	*P* value
Flat inner edge sign	0.094 (0.019–0.476)	0.004^a^	0.144 (0.024–0.876)	0.035^a^
MEPs (>50%)	26.316 (5.348–125)	<0.001^a^	18.182 (3.311–100)	<0.001^a^

OR, odds ratio; MEPs, Motor evoked potentials.

^a^ These values are significant in statistical analysis.

**Table 4:** Multivariate logistic regression analyses for estimating risk factors for paralysis.

Variables	Unadjusted OR	*P* value	Adjusted OR	*P* value
Flat inner edge sign	0.054 (0.006–0.464)	0.008^a^	0.079 (0.007–0.914)	0.042^a^
MEPs (>50%)	55.556 (8.403–333.333)	<0.001^a^	40.000 (5.236–333.333)	<0.001^a^

OE, odds ratio; MEPs, Motor evoked potentials.

^a^ These values are significant in statistical analysis.

## Discussion

We found that ischemic complications were common in insular glioma resection in the study. Additionally, patients whose tumors had the obscure inner edge and whose MEPs declined by >50% intraoperatively were at high risk for developing intraoperative strokes that resulted in motor deficits. Further, we described our strategies for arterial preservation during tumor resection.

In our patients, a transcortical approach was used for better exposure and suitability for glioma resection beyond the insula (20). The transcortical windows in our patients were limited to the pars orbitalis and triangularis of the inferior frontal gyrus. Almost all patients with postoperative speech disorders recovered their capacity for simple daily communication, suggesting that language plasticity might play a role (21). Mostly, the pars triangularis does not always involve the Broca’s area, especially for tumors located in the dominant hemispheres (10). To ensure motor function in patients with insular glioma, preservation of the arteries is critical.

The vascular supply to the insular is mainly derived from the M_2_ branches that overlie the insular surface (22). The course of the M_2_ segment along the insular surface constitutes a rich arterial network, which is a substantial obstacle in accessing the insular region (20, 23, 24), especially when the arterial network is encased in, distorted, or even wrapped around by the tumor. LLSAs may also be encased by the tumor and serve as a source of vascular supply to the tumor. The long insular artery and long medullary arteries are usually thin, long course, and have ambiguous beginnings. All these characteristics of insular gliomas make maximal resection difficult and increase the risk of ischemic events. Moreover, the insula is largely and frequently supplied by perforating arteries, with no collateral blood supply (25). New evidence suggests that motor deficits often arise from ischemia of the pyramidal tract due to compromise of the blood vessels supplying to the corona radiata and the internal capsule (18).

Moreover, most insular glioma resections affected LLSAs, long insular arteries, and even long medullary arteries terminating the M_4_ branches (3–5). Various mechanisms for developing intraoperative brain ischemia have been proposed, including direct vascular damage, vasospasm, and kinking of the arteries by brain retraction (18, 26). Therefore, preservation of these arteries is essential in insular glioma resection.

Ischemic complications of insular glioma resection with a transcortical approach are commonly detected on postoperative MRI, with incidence ranging from 1.4% to 75.4%, and are a significant source of neurological morbidity (12, 21, 26–37). The primary series of procedures for insular glioma resection using the transcortical approach are presented in Table 5. A series of 255 procedures utilizing the transcortical approach had a short-term complication rate of 12.79% and a long-term complication rate of 15.70%, 66.7% of these long-term complications could be attributed to the LLSAs and MCA infarction (30). In 61 of Pallud's 111 cases of insular gliomas who underwent awake transcortical approach, diffusion-weighted hyperintensity was observed in 39 postoperative MRI (75.4%): lenticulostriate artery territory in 5 cases, M_2_/M_3_ perforator territory in 2, and anterior choroidal artery territory in 1 (1.6%) (34). These eight deep infarcts were systematically associated with a motor deficit. In Mandonnet's series of procedures using transcortical windows for 12 patients, only 1 patient had severe dysarthria associated with a right facial palsy which resolved at 4 months; however, a slightly reduced speech rate was observed (31). Of these 12 patients, none had small areas of ischemia. In a cohort study by Przybylowski et al., which analyzed 48 patients who underwent a transcortical approach, 10 patients (21%) in the transcortical group showed evidence of ischemia on postoperative MR images (27). At the 4- to 6-week clinical evaluation, eight patients (17%) in the transcortical group demonstrated new or worsened neurological deficits. At 1 year postoperatively, four patients (8%) in the transcortical group had persistent neurological deficits. As for insular glioma surgery in this study, ischemic lesions were found in 44 (58.7%) patients using DWI, and core ischemia was noted in 11 of the 44 patients.

**Table 5:** Insular glioma resected with transcortical approach studies selected for review.

Author and year	No. of patients	Post-op DWI	Post-op transient deficits	Post-op permanent deficits
Duffau et al. 2009 (21)	24	NA	50% language deficits	NA
Sanai et al. 2010 (12)	104	NA	1.9% motor deficits 4.8% language deficits	6%
Skrap et al. 2012 (29)	66	NA	33.40%	6%
Eseonu et al. 2017 (28)	74	1.40%	8.1% motor deficits 6.8% language deficits	2.7% motor deficits
Hameed et al. 2018 (30)	255	NA	12.79%	15.70%
Berger et al. 2019 (26)	19	53%	NA	NA
Mandonnet et al. 2019 (31)	12	75%	no motor deficits 8.3% language deficits	8.3% language deficits
Przybylowski et al. 2019 (27)	48	21%	17%	8%
Leroy et al. 2021 (32)	20	NA	20%	5%
Zarino et al. 2021 (33)	32	51.4% (91.4% with transcortial)	6% language deficits	NA
Pallud et al. 2021 (34)	111	75.4% (awake resection subgroup)	awake resection subgroup (77.1%); asleep resection subgroup 42.2%,	6.25% awake resection
Rossi et al. 2021 (35)	95	20%	27.3% motor deficits 32.6% language deficits	3.2% language deficits
Panigrahi et al. 2021 (36)	23	NA	8.60%	4.3% motor deficits
Duffau 2022 (37)	5	NA	2 transitory right dysesthesia and 1 transitory phonological disorders	No

DWI, diffusion-weighted imaging; NA, not available.

Although the incidence of ischemia in our study was slightly higher than that in previous studies (26), it was still within the known range following insular glioma surgery. According to previous research, the insular tumor location is the most potent risk factor for the development of intraoperative ischemia, affecting almost all patients with ischemia (26). In our study, multivariate analysis showed that core ischemia was significantly associated with an obscure inner edge sign. This may be explained by the flat inner edge between the tumor and basal ganglia, wherein the tumor did not invade the basal ganglia; hence, the tumor could be totally resected, and the risk of LLSA damage was low. The presence of an obscure inner edge sign corresponded to a significantly increased risk of LLSA damage. In our study, four patients with less than 90% tumor removal had core ischemia. All the preoperative MRIs of these four patients showed obscure inner edge signs, which may be the main reason for core ischemia.

Tumor location and size may be a significant factor in postoperative risk, while in our study, tumor preoperative volume was not significantly associated with core ischemia, as shown in Table 1. Preoperative tumor volume was a significant predictor of EOR, which is in line with Eseonu’s study (28). Insular tumors localized in zones II and III present significant surgical difficulties, a minor extent of resection, and a higher rate of permanent morbidity (35). Also shown in our study, zone II is a major risk factor for core ischemia and paralysis.

Preservation of the LLSAs during microsurgical resection of insular gliomas is paramount. A few strategies may be effectively employed to enhance the probability of preventing iatrogenic injury to these vessels. Digital subtraction angiography was used to assess the anatomy of the vessels preoperatively (38), while indocyanine green fluorescence helped in identifying and preserving the long perforating branches of the MCA (39). Intraoperative ultrasonography may be facilitated by using contrast agents to improve LLSA visualization (40). Fluorescence-guided surgery with the use of 5-aminolevulinic acid may also help delineate the tumor at the medial edge of dissection for high-grade lesions (41). Intraoperative MRI may help assess the degree of resection and residual tumor at the medial border, which may allow further tumor resection (42). Subcortical motor mapping is performed in the medial plane of the resection to identify the internal capsule (11). However, these methods have several limitations. Notably, the number of LLSAs determined by each method is variable. Moreover, the identification of LLSAs and basal ganglia under a microscope is also essential. In combination with the surgeon’s experience, modern technology enables the safe resection of insular tumors, regardless of their anatomical and functional complexities.

Our continued experience with insular tumor resection has convinced us that the key to the resection of insular lesions is the preservation of the LLSAs and main branches of the MCA. In our experience, different strategies are used, depending on the tumor's location, according to the Berger–Sinai classification. Preservation of the proximal segment of the LLSAs in the anterior location is paramount. If the tumor encases the proximal segment of the LLSAs, it is difficult to completely resect the tumor and the risk of motor deficits increases (43). Given our experience in such situations, we made the “residual triangle” at the proximal segment of the LLSAs to preserve them. We conducted this procedure to avoid distortion, which may cause ischemia (44). In this study, the multivariate analysis did not show a significant association between the tumor encasement of the proximal segment of the LLSAs and core ischemia. Furthermore, the mean volume of the “residual triangle” was <2% of the tumor volume and had little influence on the EOR. This may confirm the effectiveness of this strategy in preserving proximal LLSAs. Moreover, the preservation of distal LLSAs is essential for tumors that are located posteriorly. Since the basal ganglia might have been invaded by the insular gliomas if there was no clear border between the tumor and basal ganglia, the “basal ganglia outline reappearance” was created based on the texture of the basal ganglia and distal branches of the LLSAs, which not only helped prevent direct injury to the posterior limb of the internal capsule but also preserved the distal branches of the LLSAs. When the texture of the basal ganglia and distal branches of the LLSAs was identified to guide the depth of resection, direct injury to the white matter motor fibers was unusual (18), and the tumor could be maximally and safely resected. Finally, in all tumor resection procedures, preservation of the main branches of M_2_ is essential. In a subpial fashion, the “sculpting” technique was used to outline the frame of the main branches of M_2_. In this way, we could cut off the insular arteries and preserve the arteries that merely passed through the tumor, i.e., the branches of M_2_.

Regarding the possible limitations of these strategies, the volume of the “residual triangle” should be left as small as possible due to the possibility of tumor relapse. Skrap et al. also advocated that they would leave a minimal part of the tumor around the LLSAs in case they were encased by the tumor (29). In the process of creating the “basal ganglia outline reappearance,” vasospasm might occur in the distal branches of the LLSAs, especially in patients with arteriosclerosis; in our study, ischemia related to LLSAs occurred in 10 patients, 7 of whose ischemia located in the head of caudate nucleus and anterior limb of internal capsule, which caused by the anterior branch of LLSAs; thus, a thin layer of the tumor or the external capsule left on the surface of basal ganglia was necessary.

While injury to the LLSAs accounts for the majority of postoperative hemiparesis following insular glioma resection (45), injury to the long insular artery is not infrequently implicated. Long insular artery and long medullary arteries terminating the M_4_ branches, which are characteristics of the thin, long course, and ambiguous origin, are difficult to be preserved intraoperatively. As in our study, eight deep-seated ischemia were caused by the long insular artery. Besides the subpial resection and transitory clipping of these vessels under MEP monitoring, Ikegaya et al. describe a strategy in which a small piece of gray matter is spared at the bottom of the peri-insular sulcus, where long insular artery and long medullary arteries pass en route to the pyramidal tract (46), which is the same principle as our “residual triangle.”

Intraoperative detection of an impending stroke is essential because a prompt response may make the decline transient instead of causing permanent deficits (26). Although intraoperative MRI is efficient in estimating the EOR, its use in detecting ischemic lesions is not recommended (47). Multiple studies verified that IONM parameters, including MEP and SSEP, are helpful and reliable for predicting and preventing ischemic brain injury during neurosurgery (48). Generally, MEP showed better diagnostic accuracy than SSEP (49). The monitoring of intraoperative MEPs revealed early ischemia and aided in preventing ischemia from becoming permanent through a therapeutic response such as holding surgery, irrigating with warm saline, and releasing retraction of the brain parenchyma (18, 50). In this study, a 50% MEP decline was significantly associated with core ischemia, postoperative paralysis, and 6 months of paralysis but not noncore ischemia. Thus, continuous IONM is essential during insular glioma resection. In our study, 4 of 11 patients with MEP abnormalities had no paralysis. Of these four patients, two with paralysis immediately after surgery recovered fully within 7 days. The other two patients may be false-positive. Abboud et al. reported a false-positive rate of 1.1% of MEPs for outcome prognostication during surgery for supratentorial lesions (51). As to 4 of 11 patients with MEP normal who had core ischemia, three of these four patients were indeed with a decline in MEP amplitude >50% intraoperatively, while withholding surgery, irrigating with warm saline, and releasing retraction of the brain parenchyma, the amplitude of these three patients recovered. Although these three patients had no paralysis, they did have core ischemia. The other patient may be with false negative, which is caused by edema in peri-resectional regions (52). After all, there is no immediate postoperative MRI scan.

Recently, awake craniotomy with intraoperative cortico-subcortical direct electrical stimulation has become an increasingly accepted and recommended technique worldwide, particularly for lesions in the dominant hemisphere (53, 54). In our study, although 4 of the 34 patients with left-sided tumors had long-term aphasia, these patients could still carry out a basic conversation. This study aimed to emphasize skill techniques for vascular preservation. If these vascular preservation strategies are applied in awake craniotomy, intraoperative direct electrical stimulation, and even network-level approaches (55), maximal insular tumor resection may be achieved, and better cognitive and emotional functional outcomes may be preserved.

### Study limitations

The main limitations of our study are its retrospective nature and small sample size. Moreover, neurological functions assessed by the neurosurgeon might leave room for observation bias. Moreover, our strategies for vascular preservation during insular glioma resection require a longer time and more cases. Finally, instead of using a rough KPS, which was the limitation of our study, a detailed description of neurocognitive and other neurological deficits, outcomes, quality of life, and daily life activities would be added in our subsequent work. The mapping of higher-order functions during the awake procedure may address these questions in the future.

## Conclusion

Insular glioma resection is associated with a high incidence of ischemia, as detected by DWI, as well as new motor deficits that were determined by the treating neurosurgeons. Insular glioma patients with obscure inner edge signs and intraoperative MEP decline >50% had a higher risk of developing core ischemia. With strategies of LLSAs and main branches of MCA preservation, such as “residual triangle” at the proximal segment of the LLSAs, “basal ganglia outline reappearance” at the distal segment of LLSAs, and “sculpting” technique for the main branches of M_2_, maximal safe resection of insular gliomas may be achieved. Nevertheless, ischemia and motor deficits occurred, even with artery-preserving strategies intended to minimize such risks. Both function and vessel protection strategies need further exploration.

## Data Availability

The original contributions presented in the study are included in the article/Supplementary Material, further inquiries can be directed to the corresponding authors.
